# Species and Sex Differences in the Morphogenic Response of Primary Rodent Neurons to 3,3′-Dichlorobiphenyl (PCB 11)

**DOI:** 10.3390/toxics6010004

**Published:** 2017-12-23

**Authors:** Sunjay Sethi, Kimberly P. Keil, Pamela J. Lein

**Affiliations:** Department of Molecular Biosciences, University of California, Davis, CA 95616, USA; sosethi@ucdavis.edu (S.S.); kpkeil@ucdavis.edu (K.P.K.)

**Keywords:** axons, dendrites, developmental neurotoxicity, in vitro, neuronal morphogenesis, PCB 11, sex bias

## Abstract

PCB 11 is an emerging global pollutant that we recently showed promotes axonal and dendritic growth in primary rat neuronal cell cultures. Here, we address the influence of sex and species on neuronal responses to PCB 11. Neuronal morphology was quantified in sex-specific primary hippocampal and cortical neuron-glia co-cultures derived from neonatal C57BL/6J mice and Sprague Dawley rats exposed for 48 h to vehicle (0.1% DMSO) or PCB 11 at concentrations ranging from 1 fM to 1 nM. Total axonal length was quantified in tau-1 immunoreactive neurons at day in vitro (DIV) 2; dendritic arborization was assessed by Sholl analysis at DIV 9 in neurons transfected with MAP2B-FusRed. In mouse cultures, PCB 11 enhanced dendritic arborization in female, but not male, hippocampal neurons and male, but not female, cortical neurons. In rat cultures, PCB 11 promoted dendritic arborization in male and female hippocampal and cortical neurons. PCB 11 also increased axonal growth in mouse and rat neurons of both sexes and neuronal cell types. These data demonstrate that PCB 11 exerts sex-specific effects on neuronal morphogenesis that vary depending on species, neurite type, and neuronal cell type. These findings have significant implications for risk assessment of this emerging developmental neurotoxicant.

## 1. Introduction

Polychlorinated biphenyls (PCBs) are ubiquitous environmental pollutants that pose a significant risk to human health [1,2,3,4]. Despite being banned from production in the late 1970s, environmental levels have not decreased significantly over the past decade [5]. This reflects the resistance of PCBs to degradation [6], the continuing release of legacy PCBs from paints and caulking in older buildings, electrical equipment, and waste disposal facilities [7,8,9,10,11,12], and the unintentional production of contemporary PCBs as byproducts of current industrial processes, such as pigment production [13]. Thus, PCBs are detected in diverse environmental and biological media ranging from water, food products, and air to human blood, brain, and placenta [14,15,16,17,18]. 

A primary endpoint of concern for human exposure to PCBs is developmental neurotoxicity. Epidemiological studies have demonstrated that PCB exposures in utero or during infancy are correlated with neurological deficits in children [1,19,20,21]. More recently, PCBs have been identified as possible risk factors for neurodevelopmental disorders (NDDs), such as autism spectrum disorder (ASD) and attention deficit hyperactivity disorder (ADHD) [22,23,24,25,26]. Research on the developmental neurotoxicity of PCBs has focused primarily on the legacy PCB congeners found in commercial PCB mixtures produced from the 1930s to 1970s. By comparison, there is negligible data regarding the developmental neurotoxicity of contemporary PCBs, many of which are lower chlorinated congeners not found in the commercial mixtures intentionally synthesized in the past [27]. These lower chlorinated PCB congeners have recently emerged as ubiquitous contaminants in various environmental media throughout the world [28,29,30,31,32,33]. Of concern, one of the more prevalent contemporary congeners, PCB 11, has recently been detected in the serum of women and their children, and in pregnant women at risk for having a child with an NDD [34,35,36]. We recently demonstrated that this pervasive pollutant interferes with neurodevelopment in vitro by promoting both axonal and dendritic growth of primary neurons in neuron-glia co-cultures comprised of cells pooled from male and female rat pups [36]. Dendritic and axonal architecture are key determinants of neuronal connectivity [37,38,39], and altered patterns of dendritic or axonal growth are implicated in the pathogenesis of various NDDs [40,41,42]. Collectively, these observations identify PCB 11 as a potential environmental risk factor for NDDs. 

Many neurological disorders, such as ASD and ADHD, exhibit a significant sex bias [43,44]. Numerous hypotheses have been advanced to explain the sex bias observed in NDDs, including sex differences in developmental rates of the brain, differential spatiotemporal patterns of hormone receptor expression in males versus females, and sex differences in innate immune responses [43,45,46]. An additional hypothesis, derived from credible evidence that many NDDs arise from complex interactions between genetic susceptibilities and environmental factors [47,48], is that the skewed sex ratios associated with various NDDs reflect sex-specific responses to environmental risk factors. Thus, a specific goal of this study was to address sex as a biological variable in the morphogenic response of primary neuron-glia co-cultures to PCB 11. Additionally, we sought to determine whether sex-dependent responses to PCB 11 vary across species. To address these critical questions, we examined the effects of PCB 11 on axonal and dendritic growth in primary neuron-glia co-cultures derived from the neocortices and hippocampi of C57BL/6J mice and Sprague Dawley rats that were separated by sex at the time of dissection to establish purified cultures of male versus female neurons. Our findings indicate that PCB 11 effects on dendritic arborization, but not axonal growth, are sex-specific but that this sex specificity varies depending on species and neuronal cell type.

## 2. Materials and Methods

### 2.1. Materials

PCB 11 (3,3′-dichlorobiphenyl) was synthesized by Dr. Xueshu Li (The University of Iowa, Iowa City, IA, USA) and confirmed to be >99% pure as determined by ^1^H NMR, ^13^C NMR, and GC-MS [36]. A MAP2B-pCAG-fusion protein red (FusRed) cDNA construct was a generous gift from Dr. Gary Wayman (University of Washington, Pullman, WA, USA) [36]. All stock solutions were made in dry, sterile dimethylsulfoxide (DMSO; Sigma-Aldrich, St. Louis, MO, USA).

### 2.2. Cell Culture

All procedures involving animals were conducted in accordance with the NIH Guide for the Care and Use of Laboratory Animals and were approved by the University of California, Davis Animal Care and Use Committee (Protocol #18813 and #18853). C57BL/6J wildtype mice were purchased from Jackson Labs (Bar Harbor, ME, USA). Female mice were paired overnight with males to obtain timed-pregnant dams. Timed-pregnant Sprague Dawley rats were purchased from Charles River Laboratory (Hollister, CA, USA). All animals were housed in clear plastic cages containing corn cob bedding under constant temperature (22 ± 2 °C) and a 12 h light-dark cycle. Food and water were provided *ad libitum*. 

Primary cortical and hippocampal neuron-glia co-cultures were prepared from postnatal day 0 mouse and rat pups as previously described [49]. The sex of each pup was determined by anogenital distance and by confirming the presence of testes for males or ovaries for females [50]. Neocortices and hippocampi from males *versus* females were separately pooled, dissociated and plated on glass coverslips (BellCo, Vineland, NJ, USA) precoated with 0.5 mg/mL poly-l-lysine (Sigma-Aldrich) and maintained at 37 °C in NeuralQ Basal Medium supplemented with 2% GS21 (MTI-GlobalStem, Gaithersburg, MD, USA) and GlutaMAX (Thermo Scientific, Waltham, MA, USA) under 5% CO_2_. Cell suspensions were plated at 83,000 cells/cm^2^ for dendritic analysis and at 33,000 cells/cm^2^ for axon quantification. Cultures used for dendritic analysis were treated with 2.5 µM cytosine β-d-arabinofuranoside (Ara-C) (Sigma-Aldrich) on day in vitro (DIV) 4 by replacing half the cellular media with medium supplemented with 5 µM Ara-C.

### 2.3. Dendritic Analyses

Peak dendritic growth occurs between DIV 4–10 in these neuronal cultures [51]. Therefore, cortical and hippocampal cultures were transfected with a MAP2B-pCAG-FusRed plasmid on DIV 6 using Lipofectamine 2000 (Invitrogen, Carlsbad, CA, USA) according to the manufacturer’s protocol. Beginning on DIV 7, cultures were exposed for 48 h to vehicle (DMSO; 1:1000) or PCB 11 diluted from 1000× stocks directly into culture medium to yield final concentrations of 1 femtomolar (fM), 1 picomolar (pM) or 1 nanomolar (nM). At the end of the exposure period, cultures were fixed in 4% paraformaldehyde (Sigma-Aldrich). Images of FusRed+ neurons were acquired using unbiased automated image acquisition software (MetaXpress Version 5.3.0.5, Molecular Devices, Sunnyvale, CA, USA, 2014) interfaced to a high content imaging system (ImageXpress, Molecular Devices). Neurons were selected for morphometric analyses using previously described criteria [52]. Briefly, these criteria included neurons whose dendritic arbor did not overlap that of adjacent neurons, dendritic tips were clearly distinguishable, and the dendritic arbor was pyramidal in shape. Dendritic arborization was quantified using Sholl plots [53] generated by ImageJ software (Version 1.49s, U.S. National Institutes of Health, Bethesda, MD, USA, 2015). The number of dendritic tips and primary dendrites was quantified manually. All dendritic analyses were performed by an individual blinded to experimental group.

### 2.4. Axonal Outgrowth

For studies of axonal growth, primary cell cultures were exposed to PCB 11 for 48 h beginning 3 h after plating. Cortical and hippocampal cell cultures were plated at a lower cell density and exposed to vehicle (DMSO; 1:1000 dilution) or PCB 11 at 1 fM, 1 pM, or 1 nM beginning 3 h post-plating for 48 h in order to visualize the complete axonal plexus of individual neurons [54]. Cortical and hippocampal cultures were immunostained for Tau-1 to visualize axons. The Tau-1 antibody used for rat cultures was from Millipore (Billerica, MA, USA); the Tau-1 antibody used for mouse cultures was from Abcam (Cambridge, MA, USA). Both antibodies were used at a 1:1000 dilution. Axonal lengths were quantified from images of Tau-1 immunopositive neurons using ImageJ software with the NeuronJ plugin [55]. As previously defined [56,57], a single neurite was considered the axon if its length was at least 2.5 times the diameter of the cell body, and it exceeded the length of all other processes extended by the same neuron. All axonal quantification was performed by an individual blinded to experimental group.

### 2.5. Statistical Analyses

All data are presented as the mean ± S.E. Statistical analyses were performed, and graphs generated, using GraphPad Prism v6.07 (San Diego, CA, USA). All experiments were performed in three to four independent dissections using three coverslips per condition per dissection (for a total of nine–12 coverslips), and the neuron was considered the statistical unit of measure. Data did not fit the assumptions for parametric analyses, therefore, data were analyzed using a nonparametric one-way ANOVA with a Dunnett’s post-hoc test; to assess sex differences, alpha was set using the Holm-Bonferroni method. 

## 3. Results

### 3.1. PCB 11 Promotes Dendritic Arborization in a Sex, Concentration and Cell Specific Manner in Mouse Neurons

**Figure 1:**
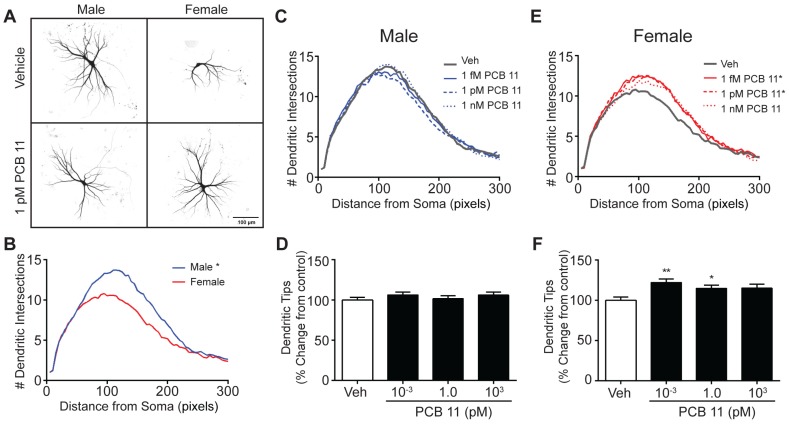
PCB 11 increases dendritic arborization in female, but not male, mouse hippocampal neurons. (**A**) Representative photomicrographs of DIV 9 FusRed+ hippocampal neurons in male and female mouse neuron-glia cocultures exposed to vehicle or 1 pM PCB 11 for 48 h. (**B**) Sholl plot illustrating sex differences in dendritic arborization in vehicle control mouse hippocampal neurons. Dendritic arborization in male (**C**,**D**) and female (**E**,**F**) mouse hippocampal neurons exposed to vehicle (0.1% DMSO) or varying concentrations of PCB 11 as quantified by Sholl analysis of dendritic complexity (**C**,**E**) and the number of dendritic tips per neuron (**D**,**F**). Data are presented as mean ± SE (*n* > 60 neurons from 3–4 independent dissections). * Significantly different from vehicle control at *p* < 0.05, ** *p* < 0.01, as determined using a nonparametric one-way ANOVA (*p* < 0.05) followed by Dunnett’s post hoc test.

Consistent with our previous observations [52], Sholl analysis of DIV 9 mouse hippocampal neurons exposed to vehicle for 48 h indicated that under our culture conditions, male neurons elaborate a more extensive dendritic arbor than female neurons (Figure 1A,B). A 48 h exposure to PCB 11 at concentrations ranging from 1 fM to 1 nM had no effect on dendritic growth in male hippocampal cultures at DIV 9 (Figure 1A,C,D). In contrast, under the same exposure conditions, PCB 11 significantly increased dendritic arborization in female hippocampal neurons at 1 fM and 1 pM, but not 1 nM. This was evidenced as an upward shift in the Sholl plot (Figure 1E) and a significantly increased number of dendritic tips per neuron (Figure 1F) for PCB 11-exposed female neurons relative to sex-matched vehicle control neurons. The number of primary dendrites per neuron for male hippocampal neurons exposed to vehicle (mean = 4.2 ± 0.12; *n* = 76 from four independent dissections) was not significantly different from that of female hippocampal neurons (mean = 4.0 ± 0.16; *n*= 61 from four independent dissections) grown under comparable conditions. Exposure to PCB 11 had no significant effect on the number of primary dendrites in male or female mouse hippocampal neurons (data not shown). 

**Figure 2:**
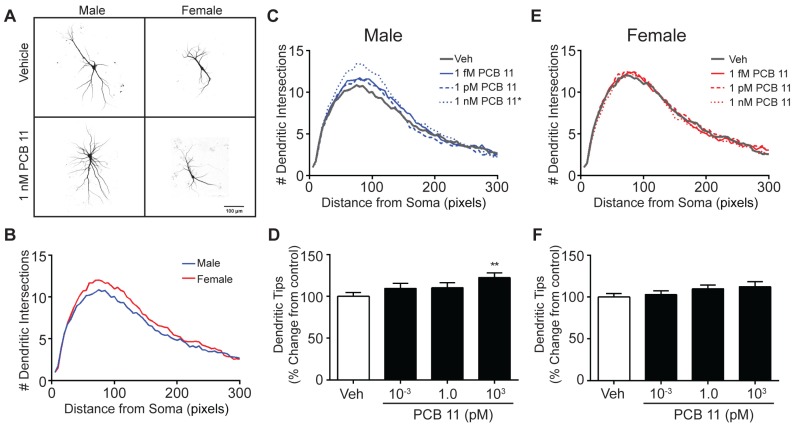
PCB 11 enhances dendritic arborization in male, but not female, mouse cortical neurons. (**A**) Representative photomicrographs of DIV 9 FusRed+ cortical neurons in male and female mouse neuron-glia cocultures exposed to vehicle or 1 nM PCB 11 for 48 h. (**B**) Sholl plot illustrating sex differences in dendritic arborization in vehicle control mouse cortical neurons. Dendritic arborization in male (**C**,**D**) and female (**E**,**F**) mouse hippocampal neurons exposed to vehicle (0.1% DMSO) or varying concentrations of PCB 11 as quantified by Sholl analysis of dendritic complexity (**C**,**E**) and the number of dendritic tips per neuron (**D**,**F**). Data are presented as mean ± SE (*n* > 80 neurons from 3–4 independent dissections). * Significantly different from vehicle control at *p* < 0.05, ** *p* < 0.01, as determined using a nonparametric one-way ANOVA (*p* < 0.05) followed by Dunnett’s post hoc test.

In contrast to mouse hippocampal neurons, there was no sex difference in the dendritic arborization of mouse cortical neurons exposed to vehicle (Figure 2A,B). However, similar to mouse hippocampal neurons, the effect of a 48 h exposure to PCB 11 on dendritic arborization in mouse cortical neurons at DIV 9 was sex-dependent, but the sex-specificity was opposite that observed for hippocampal neurons. In cortical neurons, dendritic arborization was enhanced by PCB 11 in male neurons, but only at the highest concentration tested of 1 nM (Figure 2C,D), whereas dendritic growth was not altered by PCB 11 in female neurons (Figure 2E,F). There was no significant difference in the number of primary dendrites elaborated by male (mean = 4.0 ± 0.12; *n* = 101 from four independent cultures) *versus* female (mean = 4.3 ± 0.14; *n* = 82 from four independent cultures) mouse cortical neurons; and PCB 11 did not alter the number of primary dendrites extended by cortical neurons of either sex (data not shown).

### 3.2. The Dendrite-Promoting Activity of PCB 11 Is Not Sex-Specific in Rat Neurons

**Figure 3:**
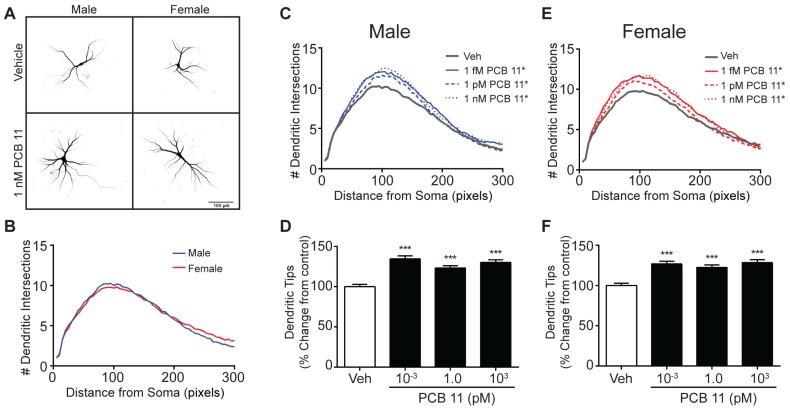
PCB 11 enhances dendritic arborization in both male and female rat hippocampal neurons. (**A**) Representative photomicrographs of DIV 9 FusRed+ hippocampal neurons in male and female rat neuron-glia cocultures exposed to vehicle or 1 nM PCB 11 for 48 h. (**B**) Sholl plot illustrating lack of sex differences in dendritic arborization in vehicle control rat hippocampal neurons. Dendritic arborization in male (**C**,**D**) and female (**E**,**F**) rat hippocampal neurons exposed to vehicle or varying concentrations of PCB 11 as quantified by Sholl analysis (**C**,**E**) and the number of dendritic tips (**D**,**F**). Data are presented as mean ± SE (*n* > 100 neurons from three–four independent dissections). * Significantly different from vehicle control at *p* < 0.05, *** *p* < 0.001 as determined using a nonparametric one-way ANOVA (*p* < 0.05) followed by Dunnett’s post hoc test.

We recently demonstrated that PCB 11 increases dendritic growth in mixed sex cultures of primary rat neurons [36], but it is unknown whether this neuronal response is sex specific. To address this question, sex separated rat neuron-glia co-cultures were exposed to vehicle or varying concentrations of PCB 11 for 48 h beginning on DIV 7. In contrast to observations of mouse hippocampal neurons, there was no sex difference in the dendritic arborization of rat hippocampal neurons exposed to vehicle (Figure 3A,B). Also unlike mouse hippocampal neurons, all concentrations of PCB 11 enhanced dendritic arborization in both male (Figure 3C,D) and female (Figure 3E,F) rat hippocampal neurons as identified by an upward shift in the Sholl plot and a significantly increased number of dendritic tips observed in PCB-exposed neurons relative to sex-matched vehicle controls. There was no significant difference in the number of primary dendrites elaborated by male (mean = 3.9 ± 0.08; *n* = 143 from three independent cultures) *versus* female (mean = 3.7 ± 0.07; *n* = 146 from three independent cultures) rat hippocampal neurons; and PCB 11 did not alter the number of primary dendrites extended by hippocampal neurons of either sex (data not shown), with one exception: at 1 nM, PCB 11 significantly increased the number of primary dendrites in female rat hippocampal neurons (mean = 4.0 ± 0.08; *n* = 141 from three independent cultures; *p* = 0.03 as determined by using a nonparametric one-way ANOVA with a Dunnett’s post-hoc).

**Figure 4:**
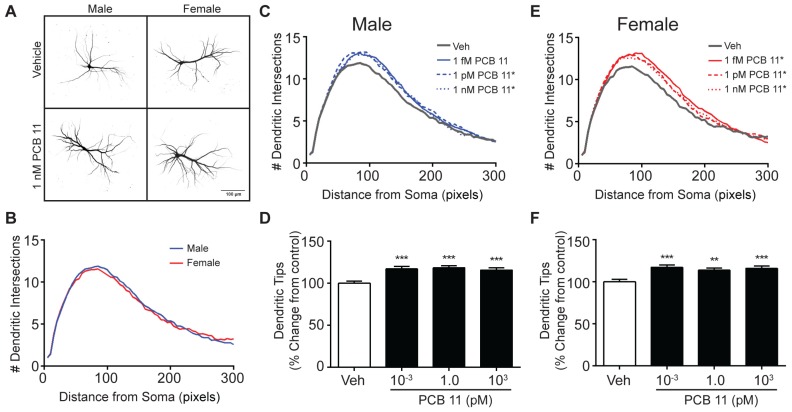
PCB 11-induced dendritic arborization in rat cortical neurons is not sex-specific. (**A**) Representative photomicrographs of DIV 9 FusRed+ cortical neurons in male and female rat neuron-glia cocultures exposed to vehicle or 1 nM PCB 11 for 48 h. (**B**) Sholl plot illustrating lack of sex differences in dendritic arborization in vehicle control rat cortical neurons. Dendritic arborization in male (**C**,**D**) and female (**E**,**F**) rat cortical neurons exposed to vehicle or varying concentrations of PCB 11 as quantified by Sholl analysis (**C**,**E**) and the number of dendritic tips (**D**,**F**). Data are presented as mean ± SE (*n* > 100 neurons from three–four independent dissections). * Significantly different from vehicle control at *p* < 0.05, ** *p* < 0.01, *** *p* < 0.001 as determined using a nonparametric one-way ANOVA (*p* < 0.05) followed by Dunnett’s post hoc test.

Similar to observations of mouse cortical neurons and rat hippocampal neurons, there were no significant sex differences in the dendritic arborization of rat cortical neurons exposed to vehicle (Figure 4A,B). However, unlike mouse cortical neurons, but similar to rat hippocampal neurons, PCB 11 promoted dendritic arborization in rat cortical neurons across the entire concentration range used in these studies in both male (Figure 4C,D) and female (Figure 4E,F) neurons as determined using Sholl analysis and by quantifying the number of dendritic tips per neuron. The number of primary dendrites did not differ significantly between male (mean = of 4.2 ± 0.09; *n* = 135 from three independent cultures) and female (mean = of 4.1 ± 0.08; *n* = 125 from three independent cultures) rat cortical neurons. PCB 11 did not change the number of primary dendrites in either male or female cortical cultures (data not shown).

### 3.3. PCB 11 Promotes Axonal Growth Independent of Sex, Cell-Type, and Species

**Figure 5:**
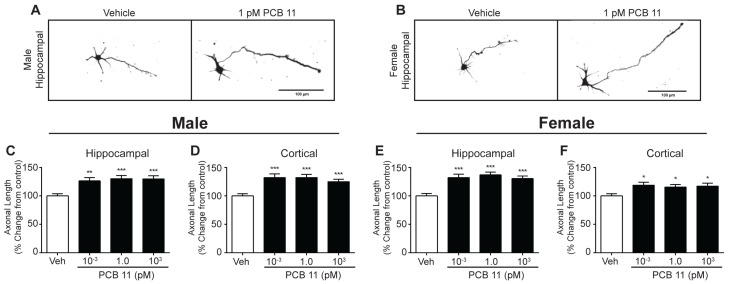
PCB 11 increases axonal growth in mouse neurons regardless of sex or cell type. (**A**,**B**) Representative photomicrographs of DIV 2 hippocampal neurons in male and female mouse neuron-glia cocultures exposed to vehicle or 1 pM PCB 11 for 48 h. Axonal length was quantified in tau-1 immunopositive male (**C**,**D**) and female (**E**,**F**) hippocampal (**C**,**E**) and cortical (**D**,**F**) mouse neurons exposed to vehicle or varying concentrations of PCB 11. Data are presented as mean ± SE (*n* = 90–100 neurons from three independent dissections). * Significantly different from vehicle control at *p* < 0.05, ** *p* < 0.01, *** *p* < 0.001, as determined using a nonparametric one-way ANOVA (*p* < 0.05) followed by Dunnett’s post hoc test.

PCB 11 has also been shown to promote axonal growth in both hippocampal and cortical neurons in mixed sex primary rat neuron-glia co-cultures [36]. To assess whether these effects are sex- and/or species-specific, sex separated cultures were set up from mice and rats and exposed to vehicle or the same concentration range of PCB 11 used for the dendritic growth studies for 48 h beginning 3 h post plating on DIV 0. In mouse hippocampal cultures, there were no significant differences in axonal growth between male (mean = 163 ± 6.3 µm; *n* = 97 from three independent cultures) and female (mean = 176 ± 7.5 µm; *n* = 92 from three independent cultures) neurons exposed to vehicle. Similarly, there was no sex difference in axonal length of mouse cortical neurons exposed to vehicle, with the mean axonal length per male and female neuron equal to 205 ± 7.8 µm and 223 ± 11.5 µm, respectively (*n* = 98–99 per group from three independent cultures). In these mouse neuron-glia co-cultures, all concentrations of PCB 11 used in these studies significantly increased axonal length per neuron in both male (Figure 5A,C,D) and female (Figure 5B,E,F) hippocampal (Figure 5A–C,E), and cortical (Figure 5D,F) neurons.

**Figure 6:**
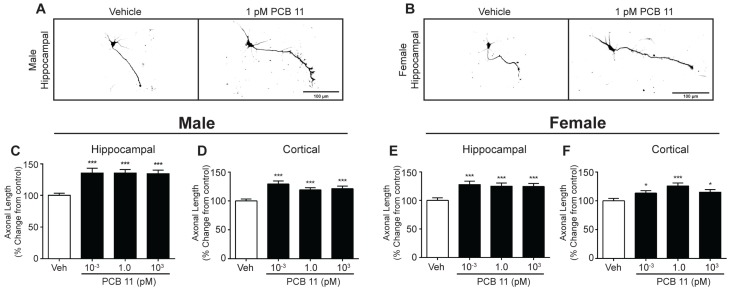
PCB 11 increases axonal growth in rat neurons regardless of sex or cell type. Representative photomicrographs of DIV 2 hippocampal neurons in male (**A**) and female (**B**) rat neuron-glia cocultures exposed to vehicle or 1 pM PCB 11 for 48 h. Axonal length was quantified in male (**C**,**D**) and female (**E**,**F**) hippocampal (**C**,**E**) and cortical (**D**,**F**) rat neurons exposed to vehicle or varying concentrations of PCB 11. Data are presented as mean ± SE (*n* = 90–100 neurons from three independent dissections). * Significantly different from vehicle control at *p* < 0.05, *** *p* < 0.001, as determined using a nonparametric one-way ANOVA (*p* < 0.05) followed by Dunnett’s post hoc test.

Similarly, there were no sex differences in axonal length in rat neurons exposed to vehicle or PCB 11. In vehicle control cultures, rat hippocampal male and female neurons had a mean axonal length of 146 ± 5.4 µm and 158 ± 7.4 µm, respectively (*n* = 86–92 per group from three independent cultures), while rat cortical male and female neurons extended axons with a mean length of 141 ± 5.0 µm and 149 ± 6.0 µm, respectively (*n* = 91–94 per group from three independent cultures). Similar to observations of mouse neurons, PCB 11 increased axon outgrowth in rat neurons of both sexes and both neuronal cell types (Figure 6).

## 4. Discussion

We recently reported that PCB 11 enhances both dendritic and axonal growth of primary rat hippocampal and cortical neurons grown in mixed sex neuron-glia co-cultures [36]. Here, we extend these previous findings by demonstrating that: (1) PCB 11 similarly promotes dendritic and axonal growth in primary mouse hippocampal and cortical neurons; (2) PCB 11 effects on dendritic arborization, but not axonal growth, are sex-specific in neurons derived from mice but not rats; and (3) the sex specificity of PCB 11’s dendrite promoting activity towards mouse neurons varies depending on the neuronal cell type. These findings are summarized in Table 1. 

A key question raised by these studies is why neuronal responses to the dendrite promoting activity of PCB 11 are sex-dependent in primary neurons derived from mice, but not rats. A plausible explanation is differential expression between mice and rats of genes that regulate dendritic growth. In support of this hypothesis, transcriptomic analyses of the dendritic structures of primary hippocampal cell cultures derived from the same mouse and rat strains used in this study revealed divergent expression of hundreds of transcripts in dendrites between mouse and rat neurons [58]. While acknowledging the potential caveat that mixed sex cultures were used for these transcriptomic analyses, these data suggest that differential gene expression between mice and rats may influence the species-specific influence of sex on dendritic responses to PCB 11. Moreover, while this study did not consider sex, it is plausible that differential expression of dendritic genes between sexes may also contribute to the sex differences seen in mice. Candidate genes include those encoding sex hormones and their cognate receptors, since the spatiotemporal expression patterns of androgen and estrogen receptors in the brain [59,60], as well as sex hormones that influence neuronal morphogenesis [61,62,63], are known to differ between mice and rats. However, data recently published by two independent laboratories demonstrated that PCB 11 has negligible activity at mammalian sex hormone receptors [64,65], which argues against this possibility. 

An alternative, but not mutually exclusive, explanation for the species-specific influence of sex on dendritic responses to PCB 11 is suggested by reports that dendritic morphogenesis and synaptogenesis occur at different rates in primary neuronal cell cultures derived from neonatal rats versus mice [66]. In vitro, mouse neurons tend to mature at a faster rate than rat neurons, with dendritic growth peaking in mouse neurons days before it does in rat neurons [66]. This raises the possibility of differing vulnerability to the dendrite promoting effects of PCB 11 based on the maturational status of the neuron and/or the rate of dendritic growth at the time of exposure. Differences in dendritic growth rates may also contribute to the differential sex specificity of PCB 11’s effects on dendritic arborization in mouse hippocampal versus cortical neurons. The developmental trajectories of the hippocampus and cortex vary according to sex. In the hippocampus, males generally tend to have a more extensive dendritic arbor than females throughout development [62,67], while in the cortex, there is a developmental window during which the dendritic arbors mature at a quicker rate and are more complex in females relative to age-matched males [68]. In agreement with these and other recently published in vivo and in vitro data [52], we observed that in primary mouse neuron-glia co-cultures, dendritic arborization is significantly more complex in male versus female hippocampal neurons, and trends towards being more complex in female versus male cortical neurons. In cultures derived from the same dissection, and analyzed at the same DIV, PCB 11 significantly enhanced dendritic growth in female but not male hippocampal neurons, and in male but not female cortical neurons. Collectively, these observations suggest that less mature neurons are more susceptible to the dendrite promoting activity of PCB 11. 

Another intriguing observation from these studies is that the sex-specific effects of PCB 11 on neuronal morphogenesis did not extend to axonal outgrowth. The axon promoting activity of PCB 11 was comparable across neuronal cell types and species. The reason(s) for the differential sex specificity of PCB 11 effects on dendrites versus axons are unclear. It is notable that while sex differences in the mammalian brain, including that of humans, are well-documented [45,69], and include sex differences in the dendritic arborization of neurons in the hippocampus and cortex of mice [52,70] and rats [68,71,72], sex differences in axonal growth are not obvious in the published literature. While this may simply reflect the fact that the influence of sex on axonal growth has not been the focus of research efforts, our observation of an apparent lack of sex-specificity in the axonal response to PCB 11 at concentrations that elicit sex-specific changes in dendritic growth suggests that PCB 11 promotes dendritic versus axonal growth via different mechanism(s). Elucidating the mechanisms of the neurite growth promoting effects of PCB 11 is the goal of future studies and will allow us to better understand the differential effects seen in axonal and dendritic growth. 

The demonstration that PCB 11 significantly alters neuronal morphogenesis in two different neuronal cell types from two different species adds to the weight of evidence suggesting that PCB 11 is a developmental neurotoxicant. Whether PCB 11 similarly interferes with dendritic and axonal growth in vivo, and whether such changes result in functional deficits, remains to be determined. However, that these in vitro data are of physiological relevance is suggested by several lines of evidence. First, altered spatiotemporal patterns of axonal or dendritic growth have been shown to cause persistent changes in brain patterning and connectivity in preclinical models [73,74,75]. Second, the female-specific effect of PCB 11 on the dendritic arborization of mouse hippocampal neurons is consistent with female-specific performance deficits on a hippocampal-dependent memory task following developmental PCB exposure [76]. Third, the in vitro morphogenic effects of PCB 11 are significant at concentrations as low as 1 fM, which are well within the range of PCB 11 levels detected in serum from women and children [34,35,36]. Considered in the context of clinical evidence linking enhanced axonal and dendritic growth, as observed in PCB 11-exposed neuron-glia co-cultures, to various NDDs, including ASD [40,41,42,77,78,79], these observations identify PCB 11 as a potential environmental risk factor for NDDs. 

An outstanding question is whether sex differences in the response of key neuronal cell populations to the developmental neurotoxicity of environmental risk factors contributes to the significant sex biases associated with many NDDs, including ASD and ADHD [43,44,80,81,82]. In support of this hypothesis, we observed that PCB 11 effects on dendritic arborization were sex-dependent in mouse neurons. However, dendritic responses of rat neurons to PCB 11 were not influenced by sex. This begs the question as to which species better models the human condition. Alternatively, if the differential sex-specific responses between neuronal cell types within mice, and between mice and rats, reflect differences in neuronal maturation at the time of exposure to PCB 11, then perhaps it is not so much the species, but rather the timing of exposure that determines whether sex influences outcome. The answers to these questions will require additional research to identify not only vulnerable windows of exposure but also the mechanism(s) by which PCB 11 interferes with neuronal morphogenesis. Nonetheless, these data provide important insights for extrapolating PCB 11 risks to the developing brain across species, sexes, and neuronal cell types.

## Figures and Tables

**Table 1:** Summary of the Axonal and Dendritic Promoting Effects of PCB 11.

	Male Mouse		Female Mouse		Male Rat		Female Rat	
	Hippocampal	Cortical	Hippocampal	Cortical	Hippocampal	Cortical	Hippocampal	Cortical
Axon	↑ All Concentrations Tested (1 fM–1 nM)							
Dendrite	-	↑ 1 nM	↑ 1 fM, 1 pM	-	↑ All Concentrations Tested			
